# Fluid shear stress regulates osteoblast proliferation and apoptosis via the lncRNA TUG1/miR‐34a/FGFR1 axis

**DOI:** 10.1111/jcmm.16829

**Published:** 2021-08-05

**Authors:** Xingwen Wang, Jinwen He, Hong Wang, Dacheng Zhao, Bin Geng, Shenghong Wang, Jiangdong An, Cuifang Wang, Hua Han, Yayi Xia

**Affiliations:** ^1^ Department of Orthopaedics Lanzhou University Second Hospital Lanzhou, Gansu China; ^2^ Orthopaedics Key Laboratory of Gansu Province Lanzhou, Gansu China

**Keywords:** apoptosis, FGFR1, fluid shear stress, lncRNA TUG1, miR‐34a, osteoblast, proliferation

## Abstract

LncRNAs and microRNAs play critical roles in osteoblast differentiation and bone formation. However, their exact roles in osteoblasts under fluid shear stress (FSS) and the possible mechanisms remain unclear. The aim of this study was to explore whether and how miR‐34a regulates osteoblast proliferation and apoptosis under FSS. In this study, FSS down‐regulated miR‐34a levels of MC3T3‐E1 cells. MiR‐34a up‐regulation attenuated FSS‐induced promotion of proliferation and suppression of apoptosis. Luciferase reporter assay revealed that miR‐34a directly targeted FGFR1. Moreover, miR‐34a regulated osteoblast proliferation and apoptosis via FGFR1. Further, we validated that lncRNA TUG1 acted as a competing endogenous RNA (ceRNA) to interact with miR‐34a and up‐regulate FGFR1 protein expression. Furthermore, lncRNA TUG1 could promote proliferation and inhibit apoptosis. Taken together, our study revealed the key role of the lncRNA TUG1/miR‐34a/FGFR1 axis in FSS‐regulated osteoblast proliferation and apoptosis and may provide potential therapeutic targets for osteoporosis.

## 1 INTRODUCTION

Mechanical load has been shown to regulate bone remodelling and homeostasis.^1^ The increase in mechanical load promotes bone formation.^2^ In contrast, the decrease of mechanical load owing to prolonged bed rest, microgravity conditions or immobilization may lead to bone loss and osteoporosis.^3^ Fluid shear stress (FSS), a form of mechanical stimulation, can activate many signal pathways and facilitate osteoblast proliferation and differentiation.^4^ Previous studies from our laboratory indicated that physiologic FSS (12 dyn/cm^2^) facilitates osteoblast proliferation through ERK5/AP‐1, Gαq/ERK5 and NFATc1/ERK5 pathways and suppresses osteoblast apoptosis through the ERK5‐AKT‐FoxO3a‐Bim/FasL pathways in MC3T3‐E1 cells.^5^, ^6^, ^7^, ^8^

As a family of single‐stranded non‐coding RNAs, microRNAs (miRNAs) post‐transcriptionally suppress gene expressions via directly targeting the 3′UTR of mRNAs.^9^, ^10^, ^11^ MiRNAs have been proved to participate in regulating bone formation.^12^ In addition, it has been shown that some miRNAs are sensitive to mechanical stimulation during osteoblast proliferation and differentiation.^13^ Our previous study demonstrated that down‐regulation of miR‐140‐5p induced by FSS could promote osteoblast proliferation by targeting VEGFA via the ERK5 pathway.^14^ Furthermore, several miRNAs have been confirmed to be involved in regulating osteoblast apoptosis, such as miR‐148a, miR‐182‐5p and miR‐543.^15^ Mai et al^16^ demonstrated that miR‐34a down‐regulation promotes osteoblast differentiation under FSS. However, whether miR‐34a could regulate proliferation and apoptosis of osteoblasts under FSS, as well as the possible mechanisms remain to be explored.

Long non‐coding RNAs (lncRNAs) are a type of transcripts that have more than 200 nucleotides and are not translated into protein.^17^ Accumulating evidence has indicated that lncRNAs regulate target genes through competitively binding to miRNAs.^18^, ^19^ LncRNAs could regulate osteogenic differentiation. For example, lncRNA MSC‐AS1 facilitated osteogenic differentiation by miR‐140‐5p.^20^ Feng et al^21^ demonstrated that linc‐ROR promoted osteogenic differentiation via sponging miR‐138 and miR‐145 in mesenchymal stem cells. However, the role of lncRNAs in osteoblasts under FSS is still unclear.

In this study, we investigated whether miR‐34a could modulate osteoblast proliferation and apoptosis under FSS and the possible mechanisms. Our results demonstrated that miR‐34a down‐regulation in response to FSS promoted proliferation and inhibited apoptosis in MC3T3‐E1 cells. Bioinformatics analysis and luciferase reporter assay revealed that FGFR1, which has been reported to be involved in regulating osteoblast proliferation and apoptosis,^22^, ^23^ was a direct target of miR‐34a. Furthermore, miR‐34a regulated osteoblast proliferation and apoptosis via FGFR1. We further revealed that lncRNA TUG1 up‐regulated FGFR1 expression by sponging miR‐34a, thereby promoting osteoblast proliferation and inhibiting osteoblast apoptosis under FSS. Our findings revealed the critical role of the lncRNA TUG1/miR‐34a/FGFR1 axis in FSS‐regulated osteoblast proliferation and apoptosis and may establish potential therapeutic strategies against osteoporosis.

## 2 MATERIALS AND METHODS

### 2.1 Cell culture

Mouse MC3T3‐E1 cells and HEK 293T cells were purchased from the Chinese Academy of Medical Sciences (Beijing, China) and were cultured in α‐MEM and DMEM with 10% FBS, respectively, at 37℃ and 5% CO_2_.

### 2.2 FSS experiment

Cells were plated on 20 × 50 mm cover slips. Then, cells were subjected to FSS (12 dyn/cm^2^) as described previously after 6 h of serum starvation.^8^ MC3T3‐E1 cells were incubated under FSS for 0, 30, 60 or 90 min.

### 2.3 Cell transfection

The transfection was performed using Lipofectamine 2000 (Invitrogen, USA) according to the manufacturer′s instructions. The transfection concentrations of mimic‐34a and inhibitor‐34a (RiboBio, China) were 50 nM and 100 nM, respectively. The FGFR1 (pcDNA3.1‐FGFR1) and lncRNA TUG1 (pcDNA3.1‐TUG1) vectors were from GenePharma (Shanghai, China). The sequences of the siRNAs for FGFR1 and lncRNA TUG1 are listed in Table S1. The concentration of plasmid was 200 ng/μL, and the siRNA concentration was 80 nM.

### 2.4 qRT‐PCR analysis

RNA was isolated by TRIzol reagent (Invitrogen, USA) according to the manufacturer's instructions. For mRNA, cDNA was synthesized using the PrimeScript^®^ RT Master Mix reagent kit (TaKaRa, Japan). Then, SYBR^®^ Premix Ex Taq^TM^ II (TaKaRa) was used to prepare qRT‐PCR on a BIO‐RAD CFX96 instrument. For miRNA, a Mir‐X miRNA First‐Strand Synthesis Kit (TaKaRa) was used to prepare the cDNA. And qRT‐PCR was then performed using SYBR^®^ Premix Ex Taq^TM^ II (TaKaRa). GAPDH or U6 was used for normalization. The primers are detailed in Table S1.

### 2.5 Luciferase assay

The sequences of wild type (WT) and mutant (MUT) for TUG1 and FGFR1 were cloned into the pmirGLO luciferase vector between the SacI and XhoI sites (GenePharma, China). Then, the FGFR1 or TUG1 vector (WT or MUT) were co‐transfected with mimic‐34a or inhibitor‐34a into 293T cells using Lipofectamine 2000 (Invitrogen, USA). Finally, the luciferase activity was examined using a luciferase assay kit (Promega, USA).

### 2.6 EdU labelling assay

Cell proliferation was evaluated using the EdU labelling kit (RiboBio, China). In brief, cells were incubated with 50 μM EdU medium diluent for 4 h. The cells were fixed with 4% paraformaldehyde for 15 min and treated with 0.5% Triton X‐100 for 20 min. Then, the cells were incubated with Apollo^®^ staining working solution for 30 min. The cells were stained with Hoechst 33342 for 30 min. Finally, the images were acquired with a fluorescence microscope (Olympus, Japan).

### 2.7 Cell counting kit‐8 assay

The cells were plated into 96‐well plates with 5 × 10^3^ cells/well. 10 μl Cell Counting kit‐8 (CCK‐8; Dojindo, Japan) was added to each well. The cells then were incubated for 3 h at 37℃. The optical density at 450 nm was analysed by an ELx800UV reader (BioTek Instruments, Winooski, VT, USA).

### 2.8 Flow cytometry

The cells were stained with an Annexin V‐FITC/PI Apoptosis Kit (BD Biosciences, USA) after resuspending in PBS according to the manufacturer's protocols. A flow cytometer was used to analyse apoptosis rates (BD Biosciences).

### 2.9 Hoechst staining

The cell nucleus was stained with Hoechst 33258 (Solarbio, China) for 15 min after 4% paraformaldehyde fixation. Finally, the nuclear morphology was observed under fluorescence microscope (Olympus, Japan).

### 2.10 RNA‐FISH assay

RNA‐FISH Cy3‐labelled lncRNA TUG1 was synthesized by RiboBio (Guangzhou, China). After 4% paraformaldehyde immobilization, the cells were prehybridized with a hybridization solution. Then, the cells were hybridized with the lncRNA TUG1 probes overnight at 37℃. Finally, the cell nucleus was labelled with DAPI. The cells were visualized under fluorescence microscope (Olympus, Japan).

### 2.11 Western blot analysis

Cells were lysed with RIPA buffer (Beyotime Biotechnology, China). Lysates were then centrifuged at 12000 rpm for 15 min, and the supernatants were collected. The extracted protein was loaded on SDS‐PAGE gels and transferred onto PVDF membranes. After blocking with skimmed milk (5%), the membranes were probed with primary antibodies including FGFR1 (1:1000, Abcam, USA), Proliferating Cell Nuclear Antigen (PCNA; 1:1000, Abcam), CDK4 (1:2000, Abcam), Cyclin D1 (1:10000, Abcam), Bax (1:1000, Cell Signaling Technology, USA), Bcl‐2 (1:1000, Cell Signaling Technology, USA), caspase‐3 (1:1000, Cell Signaling Technology, USA) and GAPDH (1:5000, Abcam) overnight at 4℃. Then, the secondary antibodies (1:1500, ZSGB‐BIO, China) were applied for 2 h. Finally, the protein bands were observed on ECL system (Bio‐Rad, USA).

### 2.12 Immunofluorescence

After 4% paraformaldehyde immobilization and 0.1% Triton X‐100 permeation, the cells were blocked with 10% normal goat serum for 30 min and incubated with primary antibody FGFR1 (1:100, Abcam) overnight at 4℃. Following Alexa Fluor 488‐conjugated secondary antibody (1:300, ProteinTech) was added, the cells were stained with DAPI and imaged using a fluorescence microscope (Olympus, Japan).

### 2.13 Statistical analysis

All data are presented as the mean ±SD of three independent experiments. Statistical significance was analysed using a two‐tailed *t* test or a one‐way ANOVA. *P*‐values <0.05 were considered statistically significant.

## 3 RESULTS

### 3.1 Down‐regulation of miR‐34a by FSS facilitates proliferation and suppresses apoptosis of osteoblasts

**FIGURE 1:**
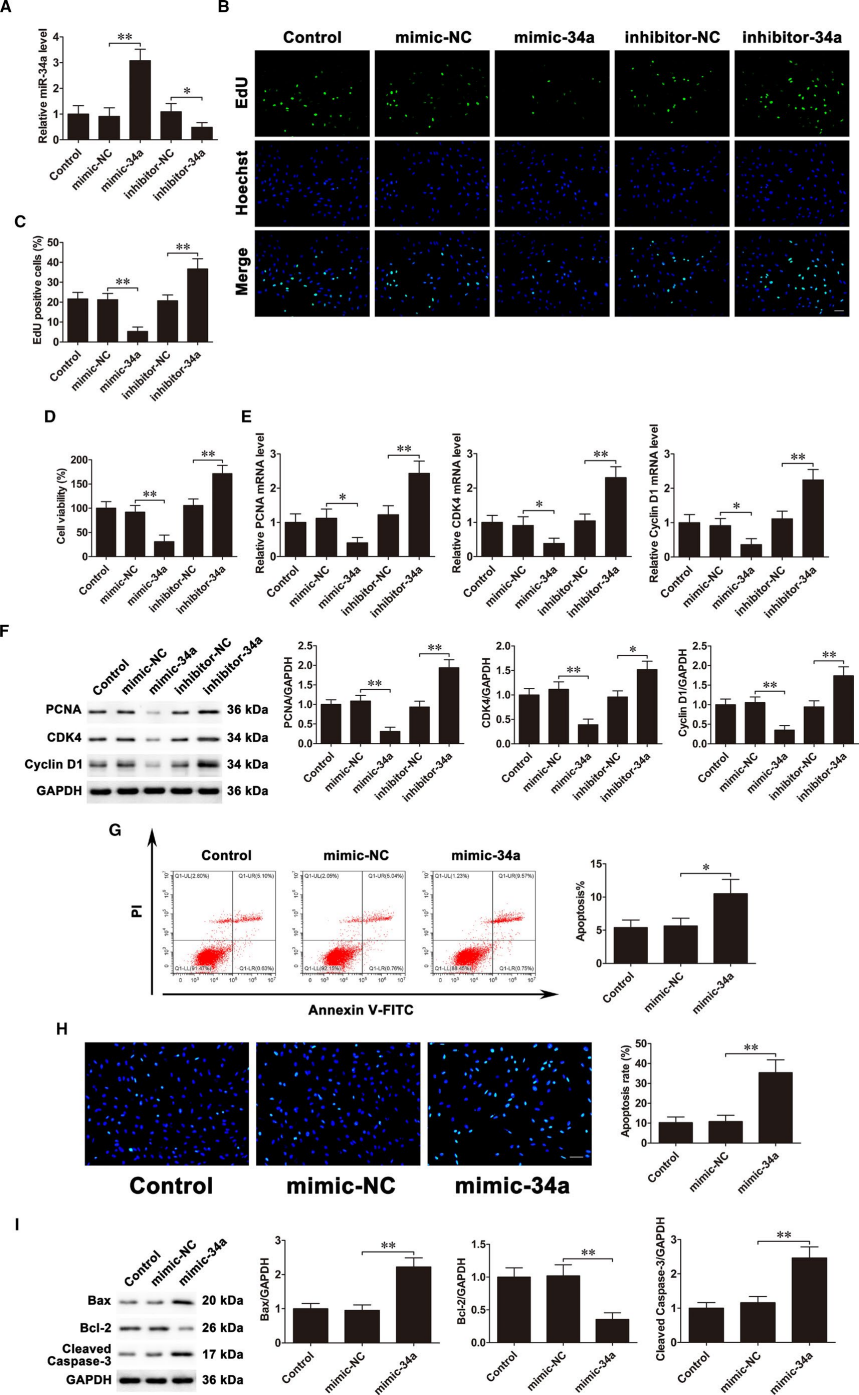
Down‐regulation of miR‐34a facilitates proliferation and suppresses apoptosis of osteoblasts. MC3T3‐E1 cells were transfected with mimic‐34a and inhibitor‐34a. A, qRT‐PCR analysis of miR‐34a expression levels. B and C, EdU assays were used to assess cell proliferation. Scale bar=50 μm. D, CCK‐8 assays examined cell proliferation. E, PCNA, CDK4 and Cyclin D1 mRNA expressions. F, PCNA, CDK4 and Cyclin D1 protein expressions. G, Flow cytometry evaluated cell apoptosis. H, Cells were stained with Hoechst. Scale bar=50 μm. I, Bax, Bcl‐2 and cleaved caspase‐3 protein expressions. Data are shown as the mean ±SD. *P < 0.05, **P < 0.01

Mimic‐34a and inhibitor‐34a were used to up‐regulate and down‐regulate the levels of miR‐34a in MC3T3‐E1 cells, respectively (Figure 1A). EdU assays and CCK‐8 assays showed that miR‐34a up‐regulation suppressed proliferation, while its down‐regulation promoted proliferation (Figure 1B‐D). We then examined the expressions of several proliferation‐associated genes. qRT‐PCR and Western blot confirmed that miR‐34a overexpression remarkably decreased the mRNA and protein expressions of PCNA, CDK4 and Cyclin D1, whereas inhibition of miR‐34a significantly increased their expressions (Figure 1E,F). Next, we assessed the influence of miR‐34a on osteoblast apoptosis. Flow cytometric analysis showed that the apoptosis rate significantly increased after mimic‐34a transfection (Figure 1G). Hoechst staining also demonstrated that up‐regulation of miR‐34a promoted cell apoptosis (Figure 1H). Furthermore, the protein expressions of several apoptosis‐related genes were evaluated by western blot. The results demonstrated that mimic‐34a transfection accelerated Bax and cleaved caspase‐3 expressions, and inhibited Bcl‐2 expression (Figure 1I). These data demonstrated that down‐regulation of miR‐34a facilitates proliferation and suppresses apoptosis of osteoblasts.

**FIGURE 2:**
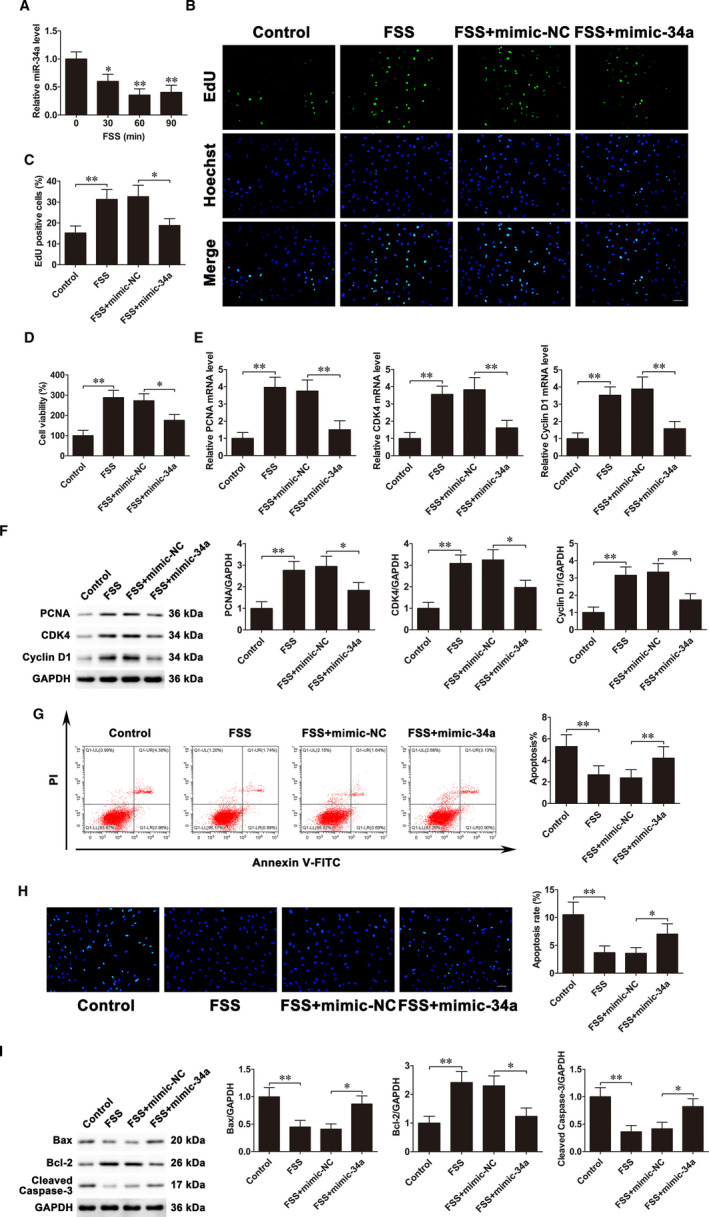
FSS down‐regulates miR‐34a expression levels, and miR‐34a up‐regulation weakens the alterations of osteoblast proliferation and apoptosis under FSS. A, qRT‐PCR analysis of miR‐34a expression levels in MC3T3‐E1 cells treated with FSS for 0 (control), 30, 60 or 90 min. B and C, MC3T3‐E1 cells were transfected with mimic‐34a before exposing to FSS. EdU assays were used to assess cell proliferation. Scale bar=50 μm. D, CCK‐8 assays examined cell proliferation. E, PCNA, CDK4 and Cyclin D1 mRNA expressions. F, PCNA, CDK4 and Cyclin D1 protein expressions. G, Flow cytometry evaluated cell apoptosis. H, Cells were stained with Hoechst. Scale bar=50 μm. I, Bax, Bcl‐2 and cleaved caspase‐3 protein expressions. Data are shown as the mean ±SD. *P < 0.05, **P < 0.01

The result of qRT‐PCR demonstrated that miR‐34a was continuously down‐regulated and reached the lowest level at 60 min under FSS (Figure 2A). Our previous studies suggested that FSS promotes osteoblast proliferation and inhibits osteoblast apoptosis.^7^, ^8^, ^24^ To investigate whether miR‐34a participates in osteoblast proliferation and apoptosis regulated by FSS, cells were transfected with mimic‐34a before exposure to FSS. EdU assays and CCK‐8 assays showed that transfection of mimic‐34a markedly inhibited proliferation induced by FSS (Figure 2B‐D). Furthermore, qRT‐PCR and Western blot revealed that miR‐34a overexpression weakened the increases of mRNA and protein levels of PCNA, CDK4 and Cyclin D1 caused by FSS (Figure 2E,F). On the other hand, flow cytometric analysis showed that mimic‐34a transfection attenuated the decrease of the apoptosis rate induced by FSS (Figure 2G). Similar trend was observed in the Hoechst staining, transfection of mimic‐34a alleviated the suppression of osteoblast apoptosis under FSS (Figure 2H). In addition, Western blot suggested that miR‐34a overexpression markedly attenuated the decreases of Bax and cleaved caspase‐3 levels and the increases of Bcl‐2 levels caused by FSS (Figure 2I). All these results suggested that down‐regulation of miR‐34a by FSS facilitates proliferation and suppresses apoptosis of osteoblasts.

### 3.2 FGFR1 mediates miR‐34a‐regulated osteoblast proliferation and apoptosis

**FIGURE 3:**
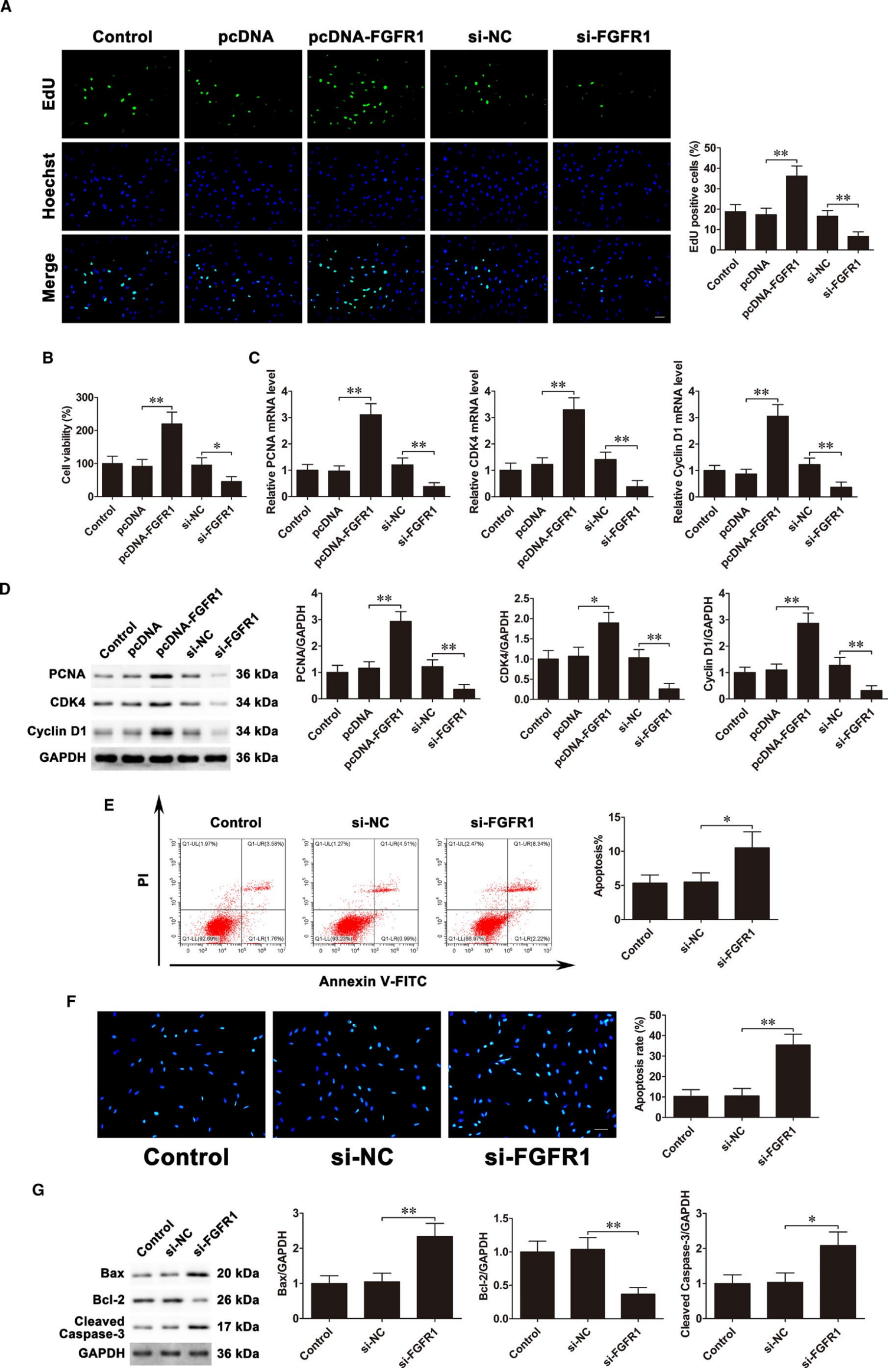
FGFR1 promotes proliferation and inhibits apoptosis of osteoblasts. pcDNA3.1‐FGFR1 and siRNA‐FGFR1 were transfected into MC3T3‐E1 cells. A, EdU assays were used to assess cell proliferation. Scale bar=50 μm. B, CCK‐8 assays examined cell proliferation. C, PCNA, CDK4 and Cyclin D1 mRNA expressions. D, PCNA, CDK4 and Cyclin D1 protein expressions. E, Flow cytometry evaluated cell apoptosis. F, Cells were stained with Hoechst. Scale bar=50 μm. G, Bax, Bcl‐2 and cleaved caspase‐3 protein expressions. Data are shown as the mean ±SD. *P < 0.05, **P < 0.01

To investigate the function of FGFR1 in osteoblast proliferation and apoptosis, we used pcDNA3.1‐FGFR1 to overexpress FGFR1 and siRNA‐FGFR1 to knockdown FGFR1 in MC3T3‐E1 cells. EdU assays and CCK‐8 assays revealed that pcDNA3.1‐FGFR1 transfection promoted cell proliferation, while siRNA‐FGFR1 transfection inhibited cell proliferation (Figure 3A,B). Moreover, the mRNA and protein expressions of PCNA, CDK4 and Cyclin D1 were accelerated in the pcDNA3.1‐FGFR1‐transfected cells, whereas considerably decreased in the siRNA‐FGFR1‐transfected cells as compared with their respective control cells (Figure 3C,D). Besides, flow cytometric analysis showed that compared with negative control, the apoptosis rate was observably up‐regulated in siRNA‐FGFR1‐transfected cells (Figure 3E). The follow‐up Hoechst staining assay revealed that FGFR1 silencing facilitated osteoblast apoptosis (Figure 3F). Western blot analysis demonstrated that FGFR1 down‐regulation led to decreased Bcl‐2 levels but elevated Bax and cleaved caspase‐3 levels (Figure 3G).

**FIGURE 4:**
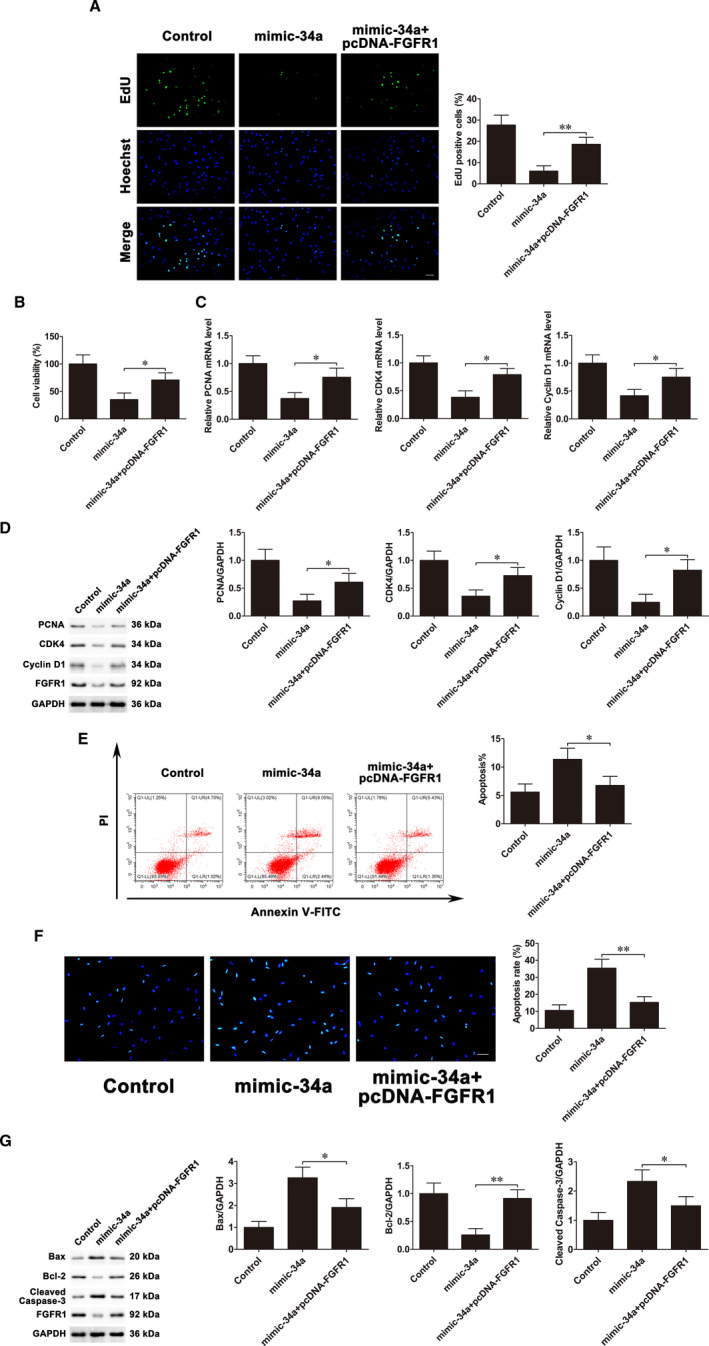
FGFR1 mediates miR‐34a‐regulated osteoblast proliferation and apoptosis. Mimic‐34a and pcDNA3.1‐FGFR1 were co‐transfected into MC3T3‐E1 cells. A, EdU assays were used to assess cell proliferation. Scale bar=50 μm. B, CCK‐8 assays examined cell proliferation. C, PCNA, CDK4 and Cyclin D1 mRNA expressions. D, PCNA, CDK4 and Cyclin D1 protein expressions. E, Flow cytometry evaluated cell apoptosis. F, Cells were stained with Hoechst. Scale bar=50 μm. G, Bax, Bcl‐2 and cleaved caspase‐3 protein expressions. Data are shown as the mean ±SD. *P < 0.05, **P < 0.01

To ascertain whether FGFR1 mediates osteoblast proliferation and apoptosis regulated by miR‐34a, mimic‐34a and pcDNA3.1‐FGFR1 were co‐transfected into MC3T3‐E1 cells. EdU assays and CCK‐8 assays revealed that miR‐34a overexpression suppressed cell proliferation, whereas co‐transfection with mimic‐34a and pcDNA3.1‐FGFR1 could counteract the suppressive effects (Figure 4A,B). Co‐transfection with mimic‐34a and pcDNA3.1‐FGFR1 partly reversed the reduction of mRNA and protein levels of PCNA, CDK4 and Cyclin D1 caused by up‐regulating miR‐34a (Figure 4C,D). In addition, the apoptosis rate was much lower in the group of co‐transfection with mimic‐34a and pcDNA3.1‐FGFR1 compared with the mimic‐34a transfection group (Figure 4E). The Hoechst staining results showed that although mimic‐34a significantly promoted apoptosis, the effects were potentially abrogated by pcDNA3.1‐FGFR1 (Figure 4F). Western blot further confirmed that pcDNA3.1‐FGFR1 dramatically weakened the increased Bax and cleaved caspase‐3 and decreased Bcl‐2 induced by miR‐34a up‐regulation (Figure 4G). These results revealed that miR‐34a played its anti‐proliferative and pro‐apoptotic roles, at least in part, through regulating FGFR1 in MC3T3‐E1 cells.

### 3.3 MiR‐34a targets FGFR1 in MC3T3‐E1 cells under FSS

**FIGURE 5:**
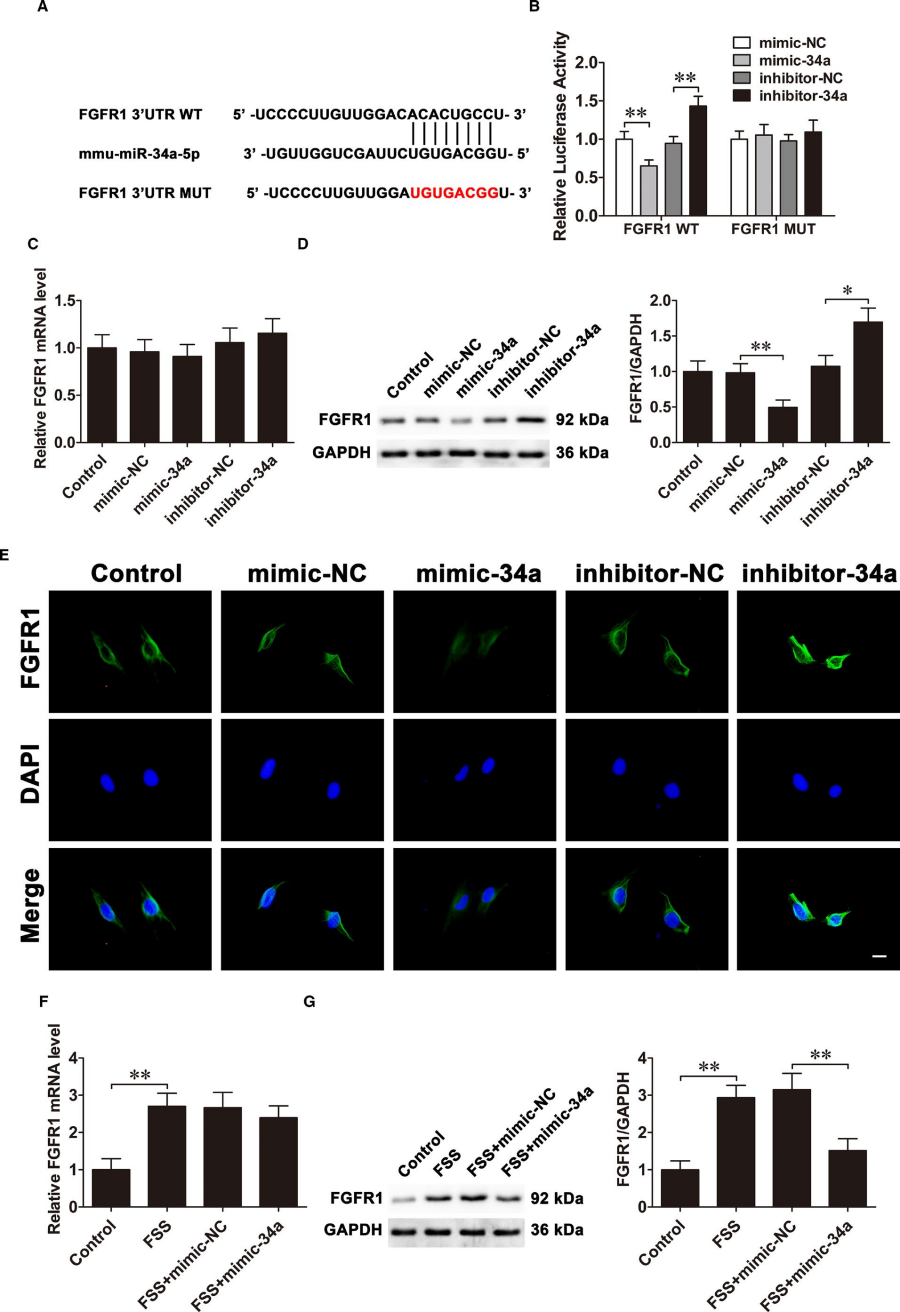
MiR‐34a targets FGFR1 in MC3T3‐E1 cells under FSS. A, The predicted miR‐34a binding sites in FGFR1 3′UTR. B, The interaction between FGFR1 and miR‐34a is identified by luciferase reporter assay. C, The FGFR1 mRNA levels in MC3T3‐E1 cells after transfection with mimic‐34a and inhibitor‐34a. D, The FGFR1 protein levels. E, Immunofluorescence analysis of FGFR1 expression levels. Scale bar =10 μm. F, The FGFR1 mRNA levels in osteoblasts transfected with mimic‐34a under FSS. G, The FGFR1 protein levels in osteoblasts transfected with mimic‐34a under FSS. Data are shown as the mean ±SD. *P < 0.05, **P < 0.01

The target genes of miR‐34a were predicted based on the TargetScan and StarBase databases. Among the many potential candidates, we focused on FGFR1, one of the FGFR family members that are involved in osteoblast proliferation and apoptosis.^22^, ^23^ To verify whether miR‐34a directly targets FGFR1, we constructed luciferase reporter vectors (WT and MUT) (Figure 5A). The luciferase reporter assay indicated that mimic‐34a obviously inhibited WT FGFR1 3′UTR luciferase activity but had no influence on that of MUT FGFR1 3′UTR. In contrary, inhibitor‐34a increased WT FGFR1 3′UTR luciferase activity but had no influence on that of MUT FGFR1 3′UTR. (Figure 5B). Western blot confirmed that alteration in miR‐34a expression mainly influenced FGFR1 protein levels, while qRT‐PCR had little effect on mRNA levels (Figure 5C,D). Immunofluorescence assay showed mimic‐34a decreased and inhibitor‐34a increased FGFR1 expression (Figure 5E). These data supported that miR‐34a targeted FGFR1.

To confirm the effects of FSS on FGFR1 expression, FSS was applied to MC3T3‐E1 cells for 1 h. qRT‐PCR and Western blot confirmed that FSS increased FGFR1 mRNA and protein levels. Furthermore, mimic‐34a inhibited only the up‐regulated FGFR1 protein levels but had little effect on the mRNA levels under FSS (Figure 5F,G). The results above illustrated that miR‐34a targeted FGFR1 in MC3T3‐E1 cells under FSS.

### 3.4 LncRNA TUG1 and miR‐34a interact with and repress each other

**FIGURE 6:**
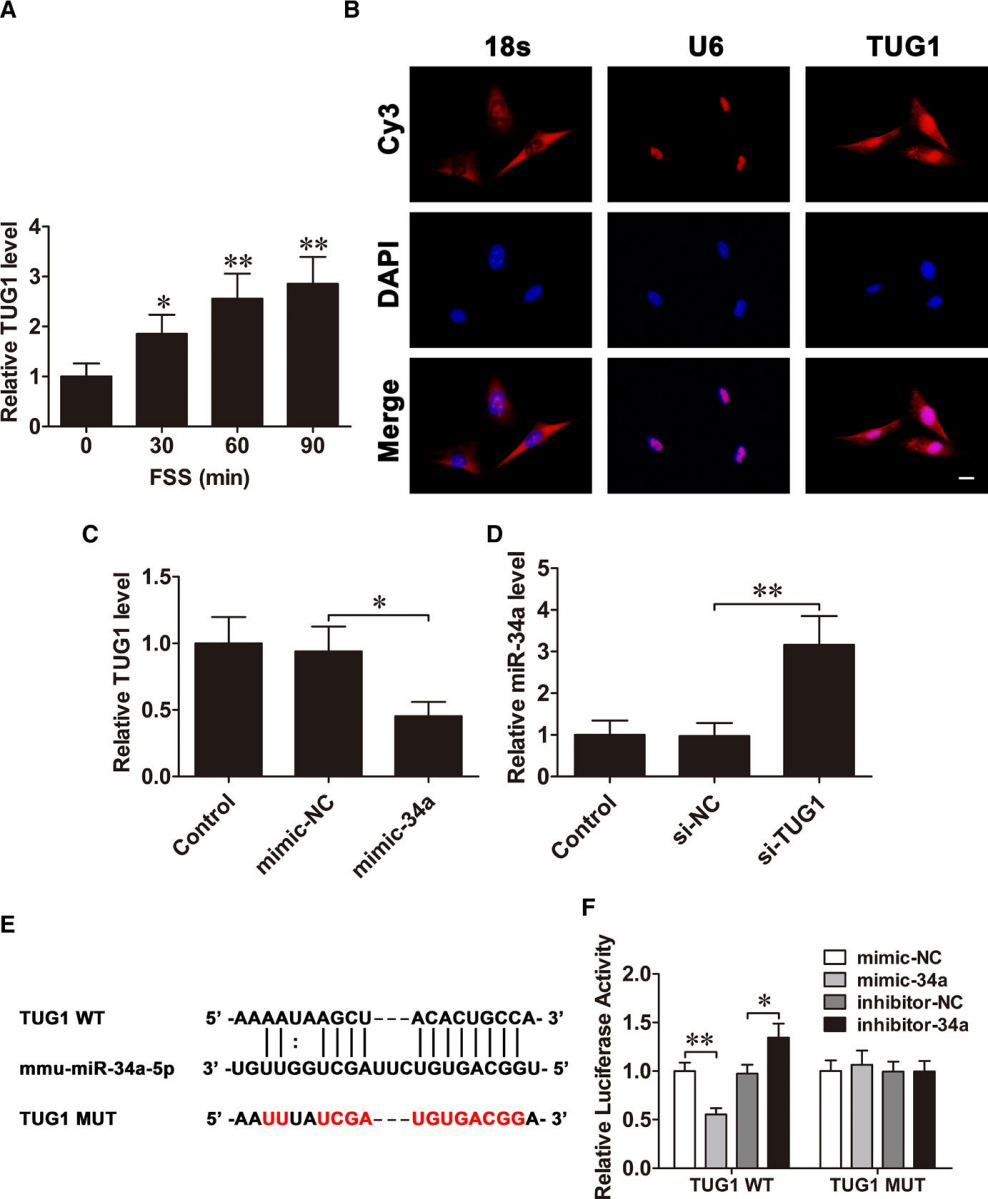
LncRNA TUG1 and miR‐34a interact with and repress each other. A, qRT‐PCR analysis of lncRNA TUG1 expression levels in MC3T3‐E1 cells treated with FSS for 0 (control), 30, 60 or 90 min. B, FISH assay analysed the cellular location of lncRNA TUG1 in osteoblasts. Scale bar=10 µm. C, The lncRNA TUG1 levels in osteoblasts after mimic‐34a transfection. D, The miR‐34a levels in osteoblasts after siRNA‐TUG1 transfection. E, The predicted miR‐34a binding sites in lncRNA TUG1. F, The interaction between lncRNA TUG1 and miR‐34a is identified by luciferase reporter assay. Data are shown as the mean ±SD. *P < 0.05, **P < 0.01

LncRNAs generally act as a ceRNA by competitively binding miRNAs, modulating target genes expression.^25^ To confirm whether miR‐34a is regulated by lncRNA, the lncRNAs that could interact with miR‐34a were predicted by StarBase database. Among the candidates, lncRNA TUG1, which is involved in regulating osteoblast proliferation and differentiation,^26^ is an important potential target. The qRT‐PCR results demonstrated that lncRNA TUG1 levels were significantly increased in response to FSS in MC3T3‐E1 cells (Figure 6A). RNA FISH analysis further confirmed that lncRNA TUG1 localized in the cytoplasm and nuclei (Figure 6B). Then, we found that up‐regulation of miR‐34a observably suppressed lncRNA TUG1 expression (Figure 6C), and knockdown of lncRNA TUG1 significantly increased miR‐34a expression (Figure 6D). To testify whether lncRNA TUG1 binds directly to miR‐34a, luciferase reporter vectors (WT and MUT) were constructed (Figure 6E). The luciferase reporter assays showed that miR‐34a up‐regulation alleviated and miR‐34a down‐regulation increased WT lncRNA TUG1 luciferase activity, but had no impact on that of MUT lncRNA TUG1 (Figure 6F).

### 3.5 LncRNA TUG1 facilitates proliferation and inhibits apoptosis of obsteoblasts under FSS through targeting miR‐34a/FGFR1 pathway

**FIGURE 7:**
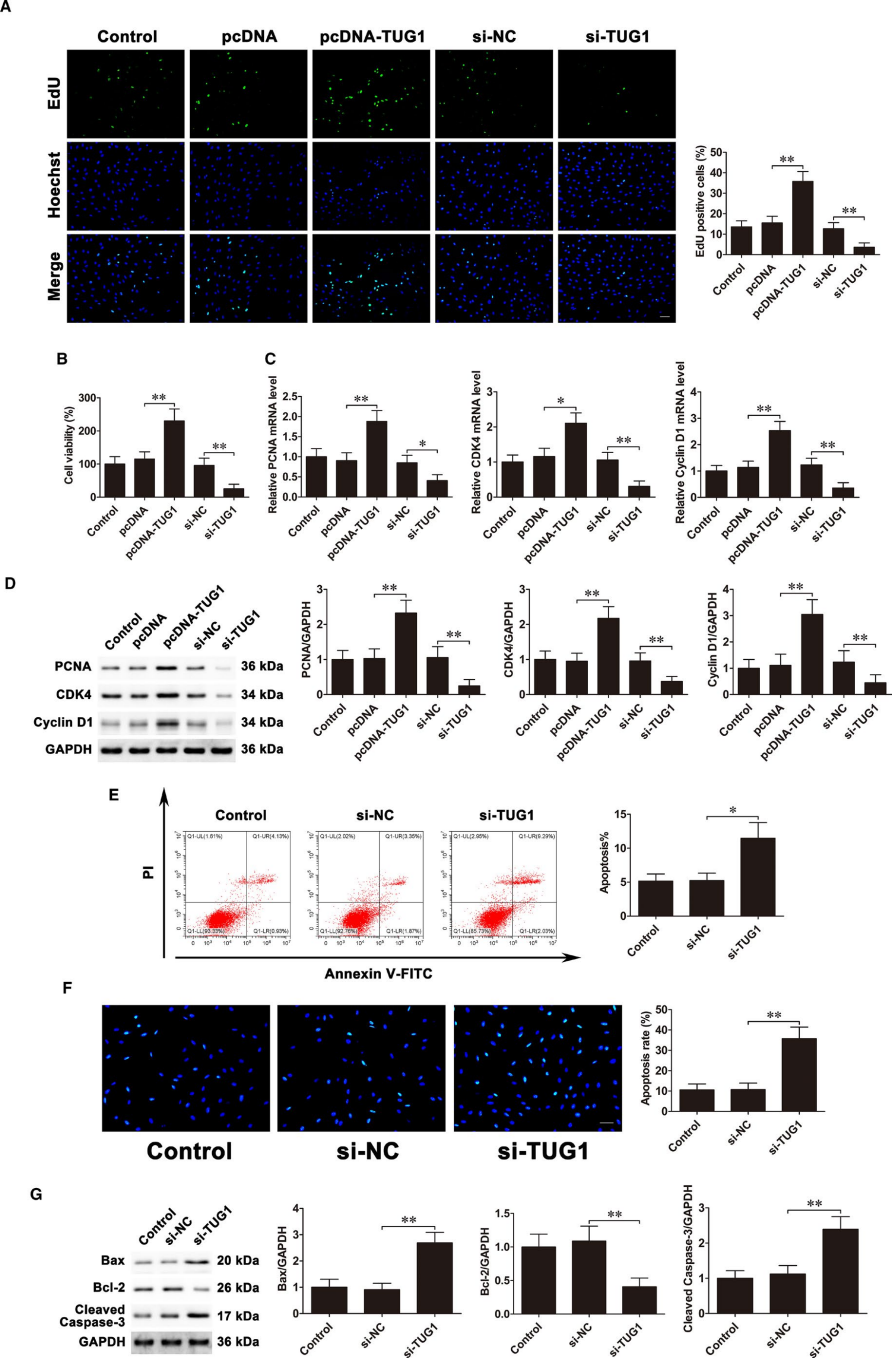
LncRNA TUG1 facilitates proliferation and inhibits apoptosis of osteoblasts. MC3T3‐E1 cells were transfected with pcDNA3.1‐TUG1 and siRNA‐TUG1. A, EdU assays were used to assess cell proliferation. Scale bar=50 μm. B, CCK‐8 assays examined cell proliferation. C, PCNA, CDK4 and Cyclin D1 mRNA expressions. D, PCNA, CDK4 and Cyclin D1 protein expressions. E, Flow cytometry evaluated cell apoptosis. F, Cells were stained with Hoechst. Scale bar=50 μm. G, Bax, Bcl‐2 and cleaved caspase‐3 protein expressions. Data are shown as the mean ±SD. *P < 0.05, **P < 0.01

LncRNA TUG1 overexpression and knockout experiments were carried out to verify the regulatory effects of lncRNA TUG1 on osteoblast proliferation and apoptosis. EdU assays and CCK‐8 assays demonstrated that overexpression of lncRNA TUG1 promoted osteoblast proliferation. Conversely, lncRNA TUG1 knockdown inhibited osteoblast proliferation (Figure 7A,B). Furthermore, the mRNA and protein expressions of PCNA, CDK4 and Cyclin D1 were detected. The results demonstrated that these genes were up‐regulated after overexpression of lncRNA TUG1. In contrast, silence of lncRNA TUG1 inhibited their expressions (Figure 7C,D). Flow cytometric analysis demonstrated that silencing lncRNA TUG1 increased cell apoptosis rate (Figure 7E). The Hoechst staining also showed siRNA‐TUG1 transfection promoted cell apoptosis (Figure 7F). The results of Western blot showed that Bax and cleaved caspase‐3 levels were increased, whereas Bcl‐2 levels were conversely decreased after down‐regulation of lncRNA TUG1 (Figure 7G).

Next, we confirmed whether lncRNA TUG1 regulates FGFR1 expression. Western blot showed that overexpression of lncRNA TUG1 markedly increased FGFR1 levels. By contrast, lncRNA TUG1 knockdown decreased FGFR1 protein expression (Figure S1A). Moreover, siRNA‐TUG1 and inhibitor‐34a were co‐transfected into MC3T3‐E1 cells before FSS exposure. Western blot results showed that transfection of inhibitor‐34a partly reversed the siRNA‐TUG1‐induced reduction in FGFR1 level under FSS (Figure S1B). Taken together, these results indicated that lncRNA TUG1 facilitates proliferation and inhibits apoptosis of obsteoblasts under FSS through targeting miR‐34a/FGFR1 pathway.

## 4 DISCUSSION

It has been documented that miRNAs participate in various biological processes, including cell proliferation, autophagy and metastasis.^27^ As a member of miRNAs, miR‐34a acts as a tumour suppressor in various human cancers, including osteosarcoma, hepatocellular carcinoma and glioblastoma. MiR‐34a was decreased in colon cancer.^28^ Liang et al^29^ confirmed that miR‐34a targeted LEF1 to inhibit metastasis of prostate cancer cells. MiR‐34a accelerated apoptosis via HMGB1 in AML cells.^30^ In addition, miR‐34a can regulate osteoblast differentiation and bone formation.^31^, ^32^ Chen et al^33^ demonstrated that down‐regulation of miR‐34a facilitated osteoblastic differentiation of hMSCs. A recent study found that miR‐34a responds to mechanical loading and plays a vital role in osteoblast differentiation.^16^ However, the role of miR‐34a in osteoblast proliferation and apoptosis under FSS is not well understood. Here, we found that miR‐34a down‐regulation in response to FSS promoted osteoblast proliferation and inhibited osteoblast apoptosis in MC3T3‐E1 cells. Moreover, we further verified that miR‐34a regulated osteoblast proliferation and apoptosis by targeting FGFR1.

FGFR1, a member of the FGFR family of receptor tyrosine kinases (RTKs), is involved in various cellular processes. When FGFR1 binds to different FGF ligands, FGFR1 can activate PKC, MAPK and PI3K/Akt pathways and participate in tumour development.^34^, ^35^, ^36^ Additionally, previous studies have indicated that FGFR1 is essential for bone development and can be modulated by miRNAs.^37^ Yang et al^38^ showed that miR‐214 inhibited osteogenic differentiation by targeting FGFR1. Here, we confirmed that FGFR1 was a direct target of miR‐34a. MiR‐34a overexpression inhibited FGFR1 protein expression, whereas miR‐34a inhibition promoted FGFR1 protein expression, which indicated that miR‐34a could negatively regulate FGFR1. It was further demonstrated that FGFR1 could promote proliferation and inhibit apoptosis of osteoblasts. The results were in accordance with previous reports in which PD166866, a selective inhibitor of FGFR1, could inhibit osteoblast proliferation in MC3T3‐E1 cells.^22^

LncRNAs could act as ceRNAs to sponge miRNAs, thus regulating osteogenic differentiation.^18^, ^39^, ^40^ However, the role of lncRNAs in osteoblasts under FSS and the possible mechanisms remain unclear. Here, we demonstrated the reciprocal regulation between miR‐34a and lncRNA TUG1, and lncRNA TUG1 was a sponge of miR‐34a. Furthermore, lncRNA TUG1 was up‐regulated in osteoblasts cultured under FSS, and lncRNA TUG1 could enhance osteoblast proliferation and inhibit osteoblast apoptosis. Hao et al^26^ reported that lncRNA TUG1 sponged miR‐545‐3p to up‐regulate CNR2, facilitating osteoblast proliferation and differentiation in hFOB1.19 cells. Liu et al^41^ discovered that lncRNA TUG1 accelerated osteoblast proliferation by Wnt pathway. Our findings are in accordance with previous conclusions that lncRNA TUG1 can indeed promote osteoblast proliferation.

**FIGURE 8:**
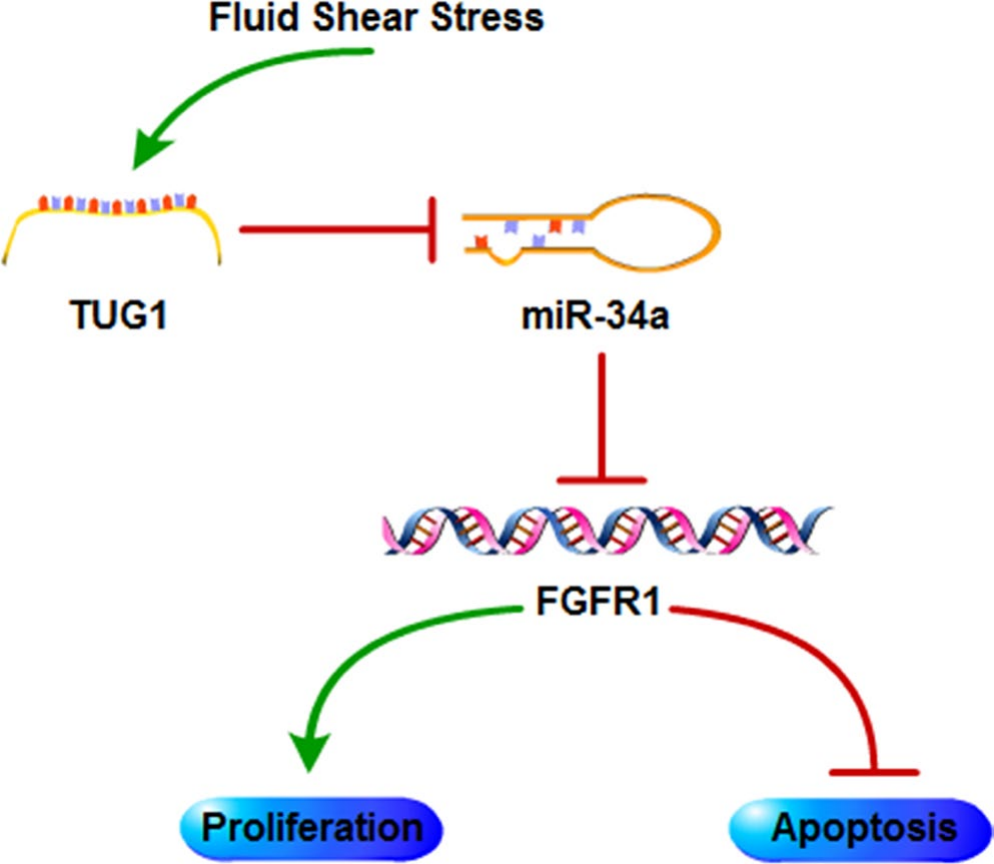
The schematic of the roles of the lncRNA TUG1/miR‐34a/FGFR1 axis in osteoblasts. MiR‐34a is down‐regulated and interacts with up‐regulated lncRNA TUG1 in osteoblasts under FSS. MiR‐34a down‐regulation promotes osteoblast proliferation and inhibits osteoblast apoptosis by targeting FGFR1

In summary, we demonstrated that miR‐34a was down‐regulated in osteoblasts in response to FSS. And down‐regulation of miR‐34a facilitated osteoblast proliferation and suppressed osteoblast apoptosis through targeting FGFR1. Moreover, lncRNA TUG1 enhanced osteoblast proliferation and suppressed osteoblast apoptosis through sponging miR‐34a to regulate FGFR1 expression (Figure 8). Therefore, our findings uncovered the role of the lncRNA TUG1/miR‐34a/FGFR1 axis in FSS‐regulated osteoblast proliferation and apoptosis and may provide promising therapeutic strategies for osteoporosis.

## CONFLICT OF INTEREST

The authors declare that they have no conflict of interest.

## AUTHOR CONTRIBUTION

**Xingwen Wang:** Conceptualization (equal); Data curation (equal); Investigation (equal); Methodology (equal); Writing‐original draft (equal). **Jinwen He:** Investigation (equal); Writing‐original draft (equal). **Hong Wang:** Data curation (equal); Methodology (equal). **Dacheng Zhao:** Investigation (equal); Visualization (equal). **Bin Geng:** Conceptualization (equal); Data curation (equal). **Shenghong Wang:** Investigation (equal); Methodology (equal). **Jiangdong An:** Formal analysis (equal); Methodology (equal). **Cuifang Wang:** Investigation (equal); Methodology (equal). **Hua Han:** Formal analysis (equal); Validation (equal). **Yayi Xia:** Conceptualization (equal); Funding acquisition (equal); Writing‐review & editing (equal).

## Supporting information

Fig S1

Fig S1 Legends

Table S1

## Data Availability

The data that support the findings of this study are available from the corresponding author upon reasonable request.

## References

[1] Hsieh YF, Turner CH. Effects of loading frequency on mechanically induced bone formation. J Bone Miner Res. 2001;16(5):918‐924. 11341337 10.1359/jbmr.2001.16.5.918

[2] Skerry TM. The response of bone to mechanical loading and disuse: fundamental principles and influences on osteoblast/osteocyte homeostasis. Arch Biochem Biophys. 2008;473(2):117‐123. 18334226 10.1016/j.abb.2008.02.028

[3] Lang T, LeBlanc A, Evans H, Lu Y, Genant H, Yu A. Cortical and trabecular bone mineral loss from the spine and hip in long‐duration spaceflight. J Bone Miner Res. 2004;19(6):1006‐1012. 15125798 10.1359/JBMR.040307

[4] Rubin J, Rubin C, Jacobs CR. Molecular pathways mediating mechanical signaling in bone. Gene. 2006;367:1‐16. 16361069 10.1016/j.gene.2005.10.028 PMC3687520

[5] Li P, Ma YC, Sheng XY, et al. Cyclic fluid shear stress promotes osteoblastic cells proliferation through ERK5 signaling pathway. Mol Cell Biochem. 2012;364(1–2):321‐327. 22286747 10.1007/s11010-012-1233-y

[6] Bo Z, Bin G, Jing W, et al. Fluid shear stress promotes osteoblast proliferation via the Galphaq‐ERK5 signaling pathway. Connect Tissue Res. 2016;57(4):299‐306. 27115838 10.1080/03008207.2016.1181063

[7] Ding N, Geng B, Li Z, et al. Fluid shear stress promotes osteoblast proliferation through the NFATc1‐ERK5 pathway. Connect Tissue Res. 2019;60(2):107‐116. 29609502 10.1080/03008207.2018.1459588

[8] Bin G, Bo Z, Jing W, et al. Fluid shear stress suppresses TNF‐alpha‐induced apoptosis in MC3T3‐E1 cells: Involvement of ERK5‐AKT‐FoxO3a‐Bim/FasL signaling pathways. Exp Cell Res. 2016;343(2):208‐217. 27060196 10.1016/j.yexcr.2016.03.014

[9] Shukla GC, Singh J, Barik S. MicroRNAs: processing, maturation, target recognition and regulatory functions. Mol Cell Pharmacol. 2011;3(3):83‐92. 22468167 PMC3315687

[10] Bartel DP. MicroRNAs: target recognition and regulatory functions. Cell. 2009;136(2):215‐233. 19167326 10.1016/j.cell.2009.01.002 PMC3794896

[11] Kosik KS. MicroRNAs and cellular phenotypy. Cell. 2010;143(1):21‐26. 20887887 10.1016/j.cell.2010.09.008

[12] Chen J, Qiu M, Dou C, Cao Z, Dong S. MicroRNAs in bone balance and osteoporosis. Drug Dev Res. 2015;76(5):235‐245. 26218893 10.1002/ddr.21260

[13] Guo Y, Wang Y, Liu Y, et al. MicroRNA‐218, microRNA‐191*, microRNA‐3070a and microRNA‐33 are responsive to mechanical strain exerted on osteoblastic cells. Mol Med Rep. 2015;12(2):3033‐3038. 25937096 10.3892/mmr.2015.3705

[14] Wang X, Geng B, Wang H, et al. Fluid shear stress‐induced down‐regulation of microRNA‐140‐5p promotes osteoblast proliferation by targeting VEGFA via the ERK5 pathway. Connect Tissue Res. 2021;1‐13. 10.1080/03008207.2021.1891228 33588662

[15] Ko NY, Chen LR, Chen KH. The role of micro RNA and long‐non‐coding RNA in osteoporosis. Int J Mol Sci. 2020;21(14):4886. 10.3390/ijms21144886 PMC7402348 32664424

[16] Mai ZH, Peng ZL, Zhang JL, et al. miRNA expression profile during fluid shear stress‐induced osteogenic differentiation in MC3T3‐E1 cells. Chin Med J (Engl). 2013;126(8):1544‐1550. 23595392

[17] Nagano T, Fraser P. No‐nonsense functions for long noncoding RNAs. Cell. 2011;145(2):178‐181. 21496640 10.1016/j.cell.2011.03.014

[18] Wang L, Wu F, Song Y, et al. Long noncoding RNA related to periodontitis interacts with miR‐182 to upregulate osteogenic differentiation in periodontal mesenchymal stem cells of periodontitis patients. Cell Death Dis. 2016;7(8):e2327. 27512949 10.1038/cddis.2016.125 PMC5108307

[19] Yuan N, Zhang G, Bie F, et al. Integrative analysis of lncRNAs and miRNAs with coding RNAs associated with ceRNA crosstalk network in triple negative breast cancer. Onco Targets Ther. 2017;10:5883‐5897. 29276392 10.2147/OTT.S149308 PMC5731337

[20] Zhang N, Hu X, He S, et al. LncRNA MSC‐AS1 promotes osteogenic differentiation and alleviates osteoporosis through sponging microRNA‐140‐5p to upregulate BMP2. Biochem Biophys Res Commun. 2019;519(4):790‐796. 31551149 10.1016/j.bbrc.2019.09.058

[21] Feng L, Shi L, Lu YF, et al. Linc‐ROR promotes osteogenic differentiation of mesenchymal stem cells by functioning as a competing endogenous RNA for miR‐138 and miR‐145. Mol Ther Nucleic Acids. 2018;11:345‐353. 29858070 10.1016/j.omtn.2018.03.004 PMC5992460

[22] Li JY, Liu SG, Xiao GN, Mao MY, Zhang XW, Sun HQ. Fibroblast growth factor receptor 1 propagates estrogen and fluid shear stress driven proliferation and differentiation response in MC3T3‐E1 cells. Mol Biol (Mosk). 2017;51(2):342‐355. 28537241 10.7868/S002689841702015X

[23] Agas D, Marchetti L, Menghi G, et al. Anti‐apoptotic Bcl‐2 enhancing requires FGF‐2/FGF receptor 1 binding in mouse osteoblasts. J Cell Physiol. 2008;214(1):145‐152. 17559077 10.1002/jcp.21170

[24] Zhang B, An L, Geng B, et al. ERK5 negatively regulates Kruppel‐like factor 4 and promotes osteogenic lineage cell proliferation in response to MEK5 overexpression or fluid shear stress. Connect Tissue Res. 2021;62(2):194‐205. 31749391 10.1080/03008207.2019.1670650

[25] Tay Y, Rinn J, Pandolfi PP. The multilayered complexity of ceRNA crosstalk and competition. Nature. 2014;505(7483):344‐352. 24429633 10.1038/nature12986 PMC4113481

[26] Hao R, Wang B, Wang H, Huo Y, Lu Y. lncRNA TUG1 promotes proliferation and differentiation of osteoblasts by regulating the miR‐545‐3p/CNR2 axis. Braz J Med Biol Res. 2020;53(11):e9798. 33053117 10.1590/1414-431X20209798 PMC7552904

[27] Fernandez‐Hernando C, Suarez Y, Rayner KJ, Moore KJ. MicroRNAs in lipid metabolism. Curr Opin Lipidol. 2011;22(2):86‐92. 21178770 10.1097/MOL.0b013e3283428d9d PMC3096067

[28] Roy S, Levi E, Majumdar AP, Sarkar FH. Expression of miR‐34 is lost in colon cancer which can be re‐expressed by a novel agent CDF. J Hematol Oncol. 2012;5:58. 22992310 10.1186/1756-8722-5-58 PMC3464169

[29] Liang J, Li Y, Daniels G, et al. LEF1 targeting EMT in prostate cancer invasion is regulated by miR‐34a. Mol Cancer Res. 2015;13(4):681‐688. 25587085 10.1158/1541-7786.MCR-14-0503 PMC4437214

[30] Liu L, Ren W, Chen K. MiR‐34a promotes apoptosis and inhibits autophagy by targeting HMGB1 in acute myeloid leukemia cells. Cell Physiol Biochem. 2017;41(5):1981‐1992. 28478444 10.1159/000475277

[31] Fan C, Jia L, Zheng Y, et al. MiR‐34a promotes osteogenic differentiation of human adipose‐derived stem cells via the RBP2/NOTCH1/CYCLIN D1 coregulatory network. Stem Cell Reports. 2016;7(2):236‐248. 27453008 10.1016/j.stemcr.2016.06.010 PMC4982986

[32] Zha X, Sun B, Zhang R, Li C, Yan Z, Chen J. Regulatory effect of microRNA‐34a on osteogenesis and angiogenesis in glucocorticoid‐induced osteonecrosis of the femoral head. J Orthop Res. 2018;36(1):417‐424. 28543623 10.1002/jor.23613

[33] Chen L, Holmstrom K, Qiu W, et al. MicroRNA‐34a inhibits osteoblast differentiation and in vivo bone formation of human stromal stem cells. Stem Cells. 2014;32(4):902‐912. 24307639 10.1002/stem.1615

[34] Thisse B, Thisse C. Functions and regulations of fibroblast growth factor signaling during embryonic development. Dev Biol. 2005;287(2):390‐402. 16216232 10.1016/j.ydbio.2005.09.011

[35] Mohammadi M, Olsen SK, Ibrahimi OA. Structural basis for fibroblast growth factor receptor activation. Cytokine Growth Factor Rev. 2005;16(2):107‐137. 15863029 10.1016/j.cytogfr.2005.01.008

[36] Brooks AN, Kilgour E, Smith PD. Molecular pathways: fibroblast growth factor signaling: a new therapeutic opportunity in cancer. Clin Cancer Res. 2012;18(7):1855‐1862. 22388515 10.1158/1078-0432.CCR-11-0699

[37] Tuzon CT, Rigueur D, Merrill AE. Nuclear fibroblast growth factor receptor signaling in skeletal development and disease. Curr Osteoporos Rep. 2019;17(3):138‐146. 30982184 10.1007/s11914-019-00512-2 PMC8221190

[38] Yang L, Ge D, Cao X, et al. MiR‐214 attenuates osteogenic differentiation of mesenchymal stem cells via targeting FGFR1. Cell Physiol Biochem. 2016;38(2):809‐820. 26872365 10.1159/000443036

[39] Liang WC, Fu WM, Wang YB, et al. H19 activates Wnt signaling and promotes osteoblast differentiation by functioning as a competing endogenous RNA. Sci Rep. 2016;6:20121. 26853553 10.1038/srep20121 PMC4745008

[40] Gu X, Li M, Jin Y, Liu D, Wei F. Identification and integrated analysis of differentially expressed lncRNAs and circRNAs reveal the potential ceRNA networks during PDLSC osteogenic differentiation. BMC Genet. 2017;18(1):100. 29197342 10.1186/s12863-017-0569-4 PMC5712120

[41] Liu SC, Sun QZ, Qiao XF, et al. LncRNA TUG1 influences osteoblast proliferation and differentiation through the Wnt/beta‐catenin signaling pathway. Eur Rev Med Pharmacol Sci. 2019;23(11):4584‐4590. 31210284 10.26355/eurrev_201906_18035

