# Supplementary figures and images for: Fluid shear stress regulates osteoblast proliferation and apoptosis via the lncRNA TUG1/miR‐34a/FGFR1 axis
Source: J Cell Mol Med. 2021 Aug 5;25(18):8734–47. doi: 10.1111/jcmm.16829 (PMC8435422; doi:10.1111/jcmm.16829)

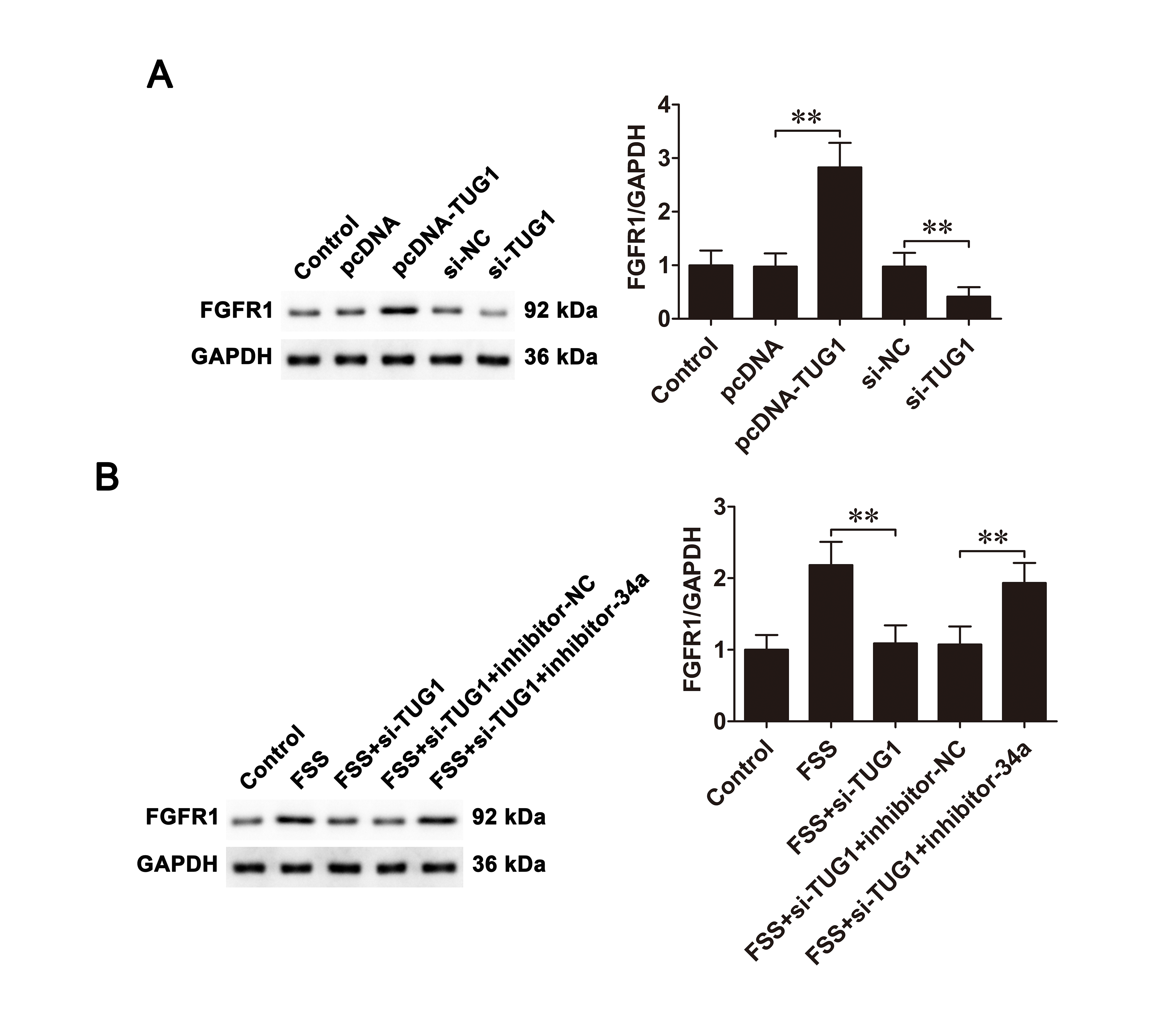

Supplement: Supplementary file 1 — Fig S1 [file JCMM-25-8734-s002.tif]
