# Supplementary material for: Fluid shear stress regulates osteoblast proliferation and apoptosis via the lncRNA TUG1/miR‐34a/FGFR1 axis
Source: J Cell Mol Med. 2021 Aug 5;25(18):8734–47. doi: 10.1111/jcmm.16829 (PMC8435422; doi:10.1111/jcmm.16829)
Supplement: Supplementary file 2 — Fig S1 Legends [file JCMM-25-8734-s003.docx]

**Supplementary Figure Legends**

**Supplementary Figure 1. LncRNA TUG1 regulates FGFR1 expression, and down-regulation of miR-34a partly reverses the siRNA-TUG1-induced reduction in FGFR1 level under FSS.** A, pcDNA3.1-TUG1, siRNA-TUG1 and their negative controls were transfected into MC3T3-E1 cells. Western blot analysis of the protein levels of FGFR1 in MC3T3-E1 cells. B, MC3T3-E1 cells were co-transfected with siRNA-TUG1 and inhibitor-34a or its negative control and then cultured under FSS. Western blot analysis of the protein levels of FGFR1 in MC3T3-E1 cells. Data are shown as the mean ± SD. ***P* < 0.01.
