# Supplementary material for: Fluid shear stress regulates osteoblast proliferation and apoptosis via the lncRNA TUG1/miR‐34a/FGFR1 axis
Source: J Cell Mol Med. 2021 Aug 5;25(18):8734–47. doi: 10.1111/jcmm.16829 (PMC8435422; doi:10.1111/jcmm.16829)
Supplement: Supplementary file 3 — Table S1 [file JCMM-25-8734-s001.docx]

**Supplementary Table 1.** The sequence of primers and siRNAs.

| Name | Sequence (5'-3') |
| --- | --- |
| PCNA-F | GAAGTTTTCTGCAAGTGGAGAG |
| PCNA-R | CAGGCTCATTCATCTCTATGGT |
| CDK4-F | GAGTGTGAGAGTTCCTAATGGA |
| CDK4-R | GGTCCTGGTCTATATGCTCAAA |
| Cyclin D1-F | CGTATCTTACTTCAAGTGCGTG |
| Cyclin D1- R | ATGGTCTCCTTCATCTTAGAGG |
| FGFR1-F | GGAGGCTACAAGGTTCGCTATGC |
| FGFR1-R | GCTGGTAGGTGTGGTTGATGCTC |
| LncRNA TUG1-F | CAAGAAACAGCAACACCAGAAG |
| LncRNA TUG1-R | TAAGGTCCCCATTCAAGTCAGT |
| GAPDH-F | GGTTGTCTCCTGCGACTTCA |
| GAPDH-R | TGGTCCAGGGTTTCTTACTCC |
| miR-34a-5p | CTGGCAGTGTCTTAGCTGGTTGT |
| U6-F | GGAACGATACAGAGAAGATTAGC |
| U6-R | TGGAACGCTTCACGAATTTGCG |
| siRNA-FGFR1 sense | GCAGCGAUACCACCUACUUTT |
| siRNA-FGFR1 antisense | AAGUAGGUGGUAUCGCUGCTT |
| siRNA-TUG1 sense | GCAGAUAUUCUGACCCAUUTT |
| siRNA-TUG1 antisense | AAUGGGUCAGAAUAUCUGCTT |
| siRNA-NC sense | UUCUCCGAACGUGUCACGUTT |
| siRNA-NC antisense | ACGUGACACGUUCGGAGAATT |
